# The Role of Motivation to Reduce Obesity among Elderly People: Response to Priming Temptation in Obese Individuals

**DOI:** 10.3390/ijerph15020244

**Published:** 2018-02-01

**Authors:** Małgorzata Obara-Gołębiowska, Hanna Brycz, Małgorzata Lipowska, Mariusz Lipowski

**Affiliations:** 1 Department of Psychology of Development and Education, University of Warmia and Mazury, Olsztyn Prawocheńskiego 13, 10-447 Olsztyn, Poland; malgorzata.obara@gmail.com; 2 Institute of Psychology, University of Gdansk, Bażyńskiego 4, 80-309 Gdansk, Poland; psyhb@ug.edu.pl; 3 Department of Health Psychology, Gdansk University of Physical Education and Sport, Górskiego 1, 80-336 Gdansk, Poland; mariusz.lipowski@awfis.gda.pl

**Keywords:** late adulthood, overweight, food related stimuli, self-regulation

## Abstract

The risk of obesity-related disorders is increased among the elderly, so changing eating habits can be an important element of prevention. The main aim of this article is to consider whether looking at pictures that present either fattening food or healthy food may motivate elderly people to change their nutrition habits. Might priming different kinds of food influence the attractiveness of the food for people in late adulthood undergoing obesity therapy? Based on priming theories, we analysed the effects of the conscious processing of stimuli associated with dietary habits in individuals aged with BMI ≥ 30 kg/m^2^. Our experiments confirmed the influence of a higher-priority goal of “slimming” on the perception and internalization of nutrition-related stimuli. In response to such stimuli, individuals who are actively involved in weight reduction and health-oriented programs use strategies for resisting temptation and to effectively “slim”. We present our findings in the context of their theoretical background and practical application.

## 1. Introduction

Overweightness and obesity have become widespread phenomena whose prevalence is increasing in most countries throughout the world [1]. Obesity can be a cause of chronic illness [2,3,4] or even death [5,6] and is observed in both rich communities and in and in those characterized by low or moderate incomes [7].

According to Eurostat research from 2017 [8], 51.6% of the European population is overweight or obese. Unfortunately in Poland, the proportion of people with obesity or overweight is 53.3% and is therefore above the average for 28 countries from the European Union [9].

Due to the physiological changes in the organism the frequency of obesity is proportionally greater among older people [10]. The increasing prevalence of overweight and obesity in elderly is especially associated with many psychophysical health problems and increased mortality. Therefore there is a need to apply various public health interventions to reduce the high prevalence of overweight and obesity among elderly population so that they can lead a socially secure life.

Many factors cause obesity. Both the roles of biophysical factors (e.g., genetics or hormones) [11,12,13], and environmental factors [14] are frequently discussed. An increasing emphasis is put on the role of changes in human lifestyle associated with modern civilisation (e.g., food environment, walkability of environs) [15], as well as psychological factors such as specific personality profiles [16,17,18], depression [19], stress [20] and, most of all, habitual patterns of behaviour [21,22]. Behavioural patterns associated with eating begin formation in earliest childhood [23] and are very difficult to change during later stages of development [24]. Not only are the roles of parents and caregivers emphasized in this process [25], but also the role of media-primarily advertising [26,27]. People watch advertisements that prime the eating of fattening food. Advertisements, even though processed on a peripheral level [28], deeply impact eating habits. However, different kinds of therapy for obese individuals seem effective in the reduction of eating tempting, delicious, and fattening food [29,30,31].

Temptations, i.e., situational counter-triggers to pursuing one’s goals (in this case the reduction of quantity of food), stimulate the development of various preventative strategies. The activation of the higher-priority goal constitutes the basis for all such strategies. By frequently repeating self-control exercises, one can develop a positive relationship between a long-term goal and a conflicting short-term goal, i.e., temptation [32]. Thus, contact with an endangering stimulus can activate the cognitive representation of a goal. This situation suggests the presence of an obstacle to long-term plans, and activates protective self-control strategies [32]. This mechanism of self-control is determined mostly by the level of involvement. It is also associated with the active pursuing of one’s priorities, which is determined by individually-developed efficient strategies of self-control [33].

The broad theoretical basis of our reasoning is the theory of goal systems [34]. Kruglanski, et al. [35] studied a group of individuals trying to lose weight. They found that priming stimuli that endangered the achievement of higher-priority goals (i.e., reducing weight) automatically induced the projection of these goals, which, in turn, caused the activation of a control mechanism preventing one from surrendering to the endangering stimulus.

Taking into account the aforementioned theoretical background and the previous experiments of Trope and Fishbach [33], Kruglanski, et al. [35], we decided to investigate our assumption about the influence of priming food-related stimuli on the improvement of dietary choices in obese individuals (BMI ≥ 30 kg/m^2^) who wish to lose weight. We decided to study the elderly population because of the prevalence of obesity in this group and the seriousness of the health consequences, like: asthma, diabetes, high cholesterol—metabolic syndrome—resulting in stroke and heart failure; as well as bone system disorder, resulting in being handicapped [36]. The identification of psychological factors related to obesity and body mass reduction allows interventions aimed at healthy ageing of elderly population. The problem is of crucial value as our population is aging in general.

In the first experiment, we verified the previously described idea of stimulating the motivation to slim through the supraliminal priming of food-related stimuli. This study was, in some way, a modified replication of Kruglanski’s experiments [35], according to whom priming increases the importance of the higher-priority goal (i.e., slimming).

The second experiment constituted an extension and partial replication of the first study. We tested the hypothesis regarding the directional effect of priming fattening or healthy food on the level of motivation to slim and the nutritional habits of obese individuals.

## 2. STUDY I: The Effect of Priming Stimuli Endangering the Goal of Slimming

### 2.1. Materials and Methods

#### 2.1.1. Objectives and Hypotheses

The aim of this study was to analyse the influence of priming fattening food on motivation to lose weight in slimming and non-slimming obese elderly. According to the theoretical background [34], an activated representation of a given higher-priority goal initiates the mechanism of preventive control, manifested by the level of importance of the related goals. The increased importance of the higher-priority goal and a higher motivation to achieve it represent a preventive strategy against surrendering to temptations [37]. This relationship applies to individuals for whom a given goal is vitally important, who are highly involved in its realization, and who have already mastered effective methods of self-control. Such a relationship could be expected in individuals who have already undergone obesity treatment. In contrast, the lack of an implemented goal should inhibit the ability to overcome temptation (i.e., it should result in a lack of self-control). We formulated the following hypothesis based on a review of the subject literature:

**Hypotheses 1** **(H1).** *The priming of fattening food in non-slimming obese individuals is associated with the decreased importance of the goal of losing weight in comparison to the increase in slimming obese participants*.

#### 2.1.2. Participants and Research Method

The study was comprised of 60 individuals with BMI ≥ 30 kg/m^2^: 32 women (age *M* = 59.36, SD = 3.63; BMI *M* = 34.5, SD = 3.08) and 28 men (age *M* = 58.25, SD = 2.43; BMI *M* = 35.4, SD = 3.15). All participants gave verbal consent to participate and the study was approved by the University of Warmia and Mazury Ethics Committee for Scientific Research, Olsztyn, Poland (ethical code number: 1/2017). The sample size of the experimental groups depended on the total number of elderly people in the obesity management clinic during the two-week weight-loss programs. All the selected participants agreed to participate in the study. Participants in this study were engaged in the experiment only once. The second study dealt with a completely different group of obese individuals. During the initial analyses, we considered age as an independent variable explaining all the other dependent variables. We found that age does not modulate any of the dependent variables, neither in interaction nor as a main effect (β non significant). We examined two gender-adjusted groups:

(1) Thirty obese individuals (BMI ≥ 30 kg/m^2^) taking part in a slimming program in the Municipal Hospital in Olsztyn (Warminsko-Mazurskie Province), Poland. All subjects had been admitted to an obesity management clinic which organizes weight loss programs which teach patients to make healthy lifestyle choices with the assistance of an interdisciplinary team of experts, including a dietician, physician, psychologist, physiotherapists and physical education trainers. All participants of the study completed all the sessions during two-week weight loss program. Among others they participated in five group and individual meetings aimed at the development of better eating self-regulatory strategies. All patients received a 1200 calorie diet. At the end of a two-week stay, patients received an individual diet and physical activity program.

(2) Thirty non-slimming obese individuals (BMI ≥ 30 kg/m^2^) from the Non-Public Health Centre in Butryny (Warminsko-Mazurskie Province in Poland), selected on the basis of their medical documentation.

All participants were investigated by means of two statements, crucial for their cognitive relation to dietary behaviour, and important for the purpose of our study as they referred to slimming, namely: (1) *Improvement of one’s silhouette*; and (2) *Losing excess weight*. Participants from both groups assessed the importance of the two dietary goals: indicated in statements using a 7-item scale, from 1—*It’s not important to me at all* to 7—*It’s of most importance for me* (dependent variable). The presented above item 1 as well as item 2 served independently as simple scales [38], each time we got two separate results that is individual answer for item 1 and item 2. The mean of item 1 and item 2 were obtained for each group to be compared. Our participants completed above two statements twice-before and after manipulation. Two weeks after the first measurement, all our subjects underwent manipulation. They were exposed to fatting food priming.

#### 2.1.3. Priming Material-Manipulation

Competent referees selected six sets of images of fattening food. All participants were presented with tempting images of fattening food. Our subjects differed in terms of their involvement in the realization of losing weight, since there were both individuals from the experimental group participating in the “slimming” program, and controls who were not undertaking any effort to reduce their weight.

#### 2.1.4. Procedure

Participants from both groups were examined individually. Initially, we kept our participants ignorant of the true objective of the study, informing them that they were to participate in an experiment determining the influence of cognitive processes on the performance of various tasks. Each participant was assigned a number during the repeated measurement of the dependent variables.

The first task pertained to completing of two items: objective 1—*Improvement of one’s silhouette*; and objective 2—*Losing excess weight* by participants from both groups. The final part of the study took place after the end of the two-week weight-loss program in the obesity clinic.

Both groups of participants were subjected to manipulation–the priming of fattening food, which according to our assumptions endangered the realization of the principal goal. The participants were presented with six sets of images of fattening food and they assessed their attractiveness and usefulness as an advertisement for a restaurant. They were asked to arrange the sets in decreasing order of attractiveness. After this exposure to fattening food stimuli, the participants performed the last task pertaining to the repeated completion of two items: objective 1—*Improvement of one’s silhouette*; and objective 2—*Losing excess weight.*

After completing the experiment, the participants were told about the real aim of the study, thanked, and fully debriefed.

#### 2.1.5. Variables

The independent variables included gender and group: 2 group (slimming obese individuals vs. non-slimming obese subjects) × 2 gender (females vs. males). Dependent variables were derived from the reproducible measurement of the motivation to realize weight loss goals. These were statements, chosen from the Goal Survey, which were considered important for the purposes of our study:

Objective 1: *Improvement of one’s silhouette*;
Objective 2: *Losing excess weight.*

### 2.2. Results

The hypothesis was verified on the basis of an analysis of variance. First of all we provided repeated analysis of variance for repeated dependent measures (the importance of two crucial goals) to verify the role of manipulation. Next, we used the design gender (2) × group (2) to explain the variance of the dependent variables (the two goals). No main gender effect of gender nor interaction gender × group were observed. Homogeneity of variance appeared to be valid, that allowed to proceed analysis of variance.

#### Verification of the Hypothesis

First of all we verified the assumption about the validation of manipulation. The analysis of variance for repeated measures counted for the first goal: the *Improvement of one’s silhouette* revealed significant effect of factor 1 (the difference between the first measurement–before manipulation and the last measurement–after the manipulation): *F* = 7.235, *p* = 0.009; and what’s of special importance: interaction effect: factor 1 × group *F* = 10.105, *p* = 0.002. Means for obese–slimming participants was: *M*_before priming_ = 5.700 vs. *M*_after priming_ = 6.440 vs. non-slimming obese participants: *M*_before priming_ = 5.112 vs. *M*_after priming_ = 4.420.

Analogues analysis provided for the goal: *Losing excess weight* showed no effect of factor 1. We found significant interaction: factor 1 × the group *F* = 5.093, *p* = 0.028. Means for obese slimming participants: *M*_before priming_ = 6.005 vs. *M*_after priming_ = 6.444 versus non-slimming obese participants: *M*_before priming_ = 3.459 vs. *M*_after priming_ = 3.850.

The priming worked according to the hypothesis of counteractive control [34] for slimming individuals whereas the same priming provoked non-slimming participants to lower the importance of goal “loosing excess weight”. Manipulation check is in concordance with our hypothesis: the priming of fattening food should result in the diminished importance of the goals of losing weight and attaining a good silhouette, or loosing kilograms in non-slimming obese individuals in comparison to the importance of these same goals among slimming obese individuals.

**Figure 1:**
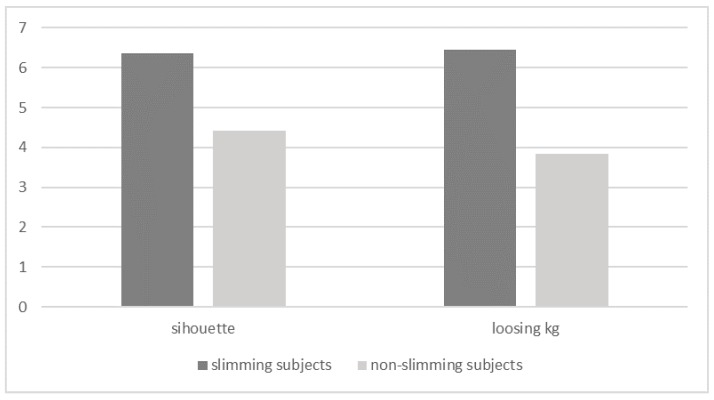
The importance of dietary goals as a result of priming among sliming vs. non-slimming subjects.

Moreover, we expect that slimming individuals assign higher importance to two goals then non-slimming participants. It appeared to be the truth (see Figure 1).

In other words, we expected that the importance of the two goals would differ significantly between the last measure of goals (after priming) two groups of individuals.

In the case of the *Improvement of one’s silhouette* goal we found:

- A significant effect of the group: *F*(1.56) = 28.22, *p* < 0.001; *eta*^2^ = 0.34, *M* = 6.37 vs. *M* = 4.42, *t*(58) = 5.38, *p* < 0.001. This means, as shown in Figure 1, that as a result of priming fattening food, slimming obese individuals paid more attention to the realization of the *Improvement of one’s silhouette* goal than did non-slimming obese individuals.

In the case of the *Losing excess weight* we observed:

- A significant effect of the group: *F*(1.56) = 48.41, *p* < 0.001, *eta*^2^ = 0.462, *M* = 6.44 vs. *M* = 3.85, *t*(58) = 6.76, *p* < 0.001.

As with the previously analysed goal, losing excess weight proved more important in the case of slimming obese individuals than in that of non-slimming individuals (Figure 1).

### 2.3. Discussion

The aim of the study was to verify if priming fattening food in slimming and non-slimming obese individuals would be reflected in an activation of the mental representation of the goal (i.e., slimming), in turn activating preventive control, and influencing the motivational aspect of self-regulation. Our hypothesis assumed that priming fattening food resulted in higher importance being attached to losing weight for slimming obese individuals than for non-slimming individuals. This hypothesis was confirmed with regards to both dependent variables. We found that individuals who were actively slimming attached more importance to the two goals (*improvement of one’s silhouette* and *losing excess weight*) in comparison to obese persons who were not attempting to lose weight. The literature suggests that the number of kilograms lost is the most important index of successful treatment outcome, followed by the decrease of body circumference in centimeters, and the improvement in one’s silhouette. However, a high number of kilograms lost is not always an objective measure of successful slimming as, not infrequently, physical exercise included in the obesity treatment program results in the replacement of adipose tissue with muscle tissue without a significant reduction in body weight [39,40]. Therefore, due to different physiological conditions, obese individuals participating in the program may pay greater attention to other aspects of the slimming process. Consequently, the aforementioned indices should be considered equally important.

According to previously published theories, individuals who are motivated and involved in achieving a given goal utilize anti-temptation strategies [33,37,41]. The situation is different in persons who are not motivated to slim. Priming fattening food led to decreased importance being attached to the *improvement of one’s silhouette* as well as the *losing excess weight* goals in this group.

We expected such an outcome, as according to the theoretical background there are two prerequisites of an asymmetrical facilitating association between the risk and the higher-priority goal, which activates control measures preventing the surrender to temptation. Firstly, individuals must be highly motivated and involved in the realization of the long-term goal, as well as to be diligent. Secondly, they must use individually developed, extensively and repeatedly practiced efficient strategies of self-control to enable them to achieve their goals [37]. While this first condition is definitely satisfied in the case of slimming obese individuals, the second one is apparently not-as BMI ≥ 30 kg/m^2^ suggests that these individuals did not learn efficient strategies for maintaining normal body weight. However, it should be noted that this quasi-experiment took place at the end of the slimming program, when the obese subjects were still learning and practicing effective strategies of dietary control under the supervision of specialists. Therefore, individuals from this group developed an asymmetrically facilitating relationship between their goal and the endangering temptation. Looking at fattening food did not lead to the abandoning of the goal of slimming as was observed in the non-slimming group. Motivation, involvement in the realization of the higher-priority goal, and acquired and practiced strategies of self-control prevented the diminishing of importance of the higher-priority goal. Consequently, the temptation associated with fattening food did not endanger the goal of slimming.

## 3. STUDY II: The Influence of Priming Stimuli Endangering or Consistent with the Higher-Priority Goal (Slimming), and Its Impact on Behaviour Dependent Measures

### 3.1. Materials and Methods

#### 3.1.1. Objectives and Hypotheses

Study II was an extended version of Study I. The aim of this study was to analyse the effect of priming fattening or diet food in obese elderly individuals with an implemented goal, i.e., on the motivational and behavioural aspects of self-regulation related to losing weight in slimming subjects. We verified if the level of motivation and practice of effective self-control techniques of obese individuals was sufficient to develop the asymmetrically facilitating relationship between stimuli which are consistent or inconsistent with the goal of losing weight. Consequently, we verified if priming fattening food would induce a mental representation of the higher-priority goal, i.e., slimming. According to our assumptions, the development of such an association should be reflected by the inducing of preventive control, protecting against the temptation of fattening food [35]. Moreover, we verified whether priming healthy food motivated our participants to slim and engage in other behaviours consistent with the higher-priority goal of losing weight. The true behaviour of obese individuals was the core of the experiment.

The following hypotheses were formulated:

**Hypotheses 2** **(H2).** *On priming with healthy food, obese individuals choose healthy refreshments more frequently than do the controls*.

**Hypotheses 3** **(H3).** *On priming with fattening food, obese individuals choose healthy refreshments more frequently than do the controls*.

#### 3.1.2. Participants and Research Methods

The study included 102 obese individuals (BMI ≥ 30 kg/m^2^): 56 women (age *M* = 58.51, *SD* = 3.24), BMI *M* = 35.5 (*SD* = 3.12) and 48 men (age *M* = 59.34, *SD* = 3.46), BMI *M* = 35.8 (*SD* = 2.9). All subjects had been admitted to an obesity management clinic which organizes weight loss programs which teach patients to make healthy lifestyle choices with the assistance of an interdisciplinary team of experts, including a dietician, physician, psychologist, physiotherapists and physical education trainers. All the selected participants agreed to participate in the study. They gave verbal consent to participate and the study was approved by the University of Warmia and Mazury Ethics Committee for Scientific Research, Olsztyn, Poland (ethical code number: 1/2017).

The experiment included three groups (each *n* = 34) of obese individuals: (1) an experimental group subjected to priming for fattening food, (2) an experimental group subjected to priming for healthy food, and (3) a control group subjected to neutral priming with images unrelated to slimming (landscapes, pictures of nature, etc.). Participants were randomly assigned to experimental groups.

We used images of fattening and healthy food, as well as sets of neutral images (unrelated to food).

Experimental group 1 was primed for fattening food with the sets of images of fattening food used previously in Study I.

Experimental group 2 was primed for healthy food with six sets of images, each containing six images of healthy-looking food. The appropriateness of these images was also tested for this study by competent referees.

The control group was subject to neutral priming with six sets of images, each containing six neutral images completely unrelated to food or slimming, such as landscapes or pictures of nature.

The independent variables included gender and group: (1) experimental group: priming fattening food; (2) experimental group: priming diet food; and (3) control group: neutral priming.

The dependent variable was a behavioural one. After the experiment, each participant chose a snack: healthy or fattening. The choice of healthy versus fattening snack was based on the selection of one food product from a list containing items such as chocolate, apples, carrots, cakes, grapefruits and crisps. The calorie content of these products was determined with the use of relevant reference tables.

#### 3.1.3. Procedure

Obese individuals participating in the program (i.e., those with the goal of slimming) were examined individually. The experiment took place after the end of the two-week weight-loss program in the obesity management clinic.

Initially, as in Study I, we kept our participants ignorant of the true objective of the study, informing them that they were participating in an experiment determining the influence of cognitive processes on performing various tasks. Each participant was assigned a number during the repeated measurement of the dependent variables.

Experimental group 1 was primed for fattening food, which, according to our assumption, endangered the realization of the higher-priority goal (slimming). As in Study I, the participants were presented with six sets of images of fattening food and assessed their attractiveness and usefulness as an advertisement for a restaurant. They were asked to arrange the sets in decreasing order of attractiveness. The aim of this task was to expose the participants to a strong food stimulus, while intensively involving cognitive processes.

Experimental group 2 was primed for healthy food, which, according to our assumption, supported the realization of the higher-priority goal, i.e., slimming. The participants were presented with six sets of images of diet food and assessed their attractiveness and usefulness as an advertisement for a restaurant serving low-calorie food. They were asked to arrange the sets in decreasing order of attractiveness. The aim of this task was to expose the participants to a strong healthy food stimulus, while intensively involving cognitive processes.

The controls were subject to neutral priming. The participants were presented with six sets of various images of nature and asked to assess their visual attractiveness. They were asked to arrange the sets in decreasing order of attractiveness. The aim of this task was to expose the participants to neutral stimuli completely unrelated to food or slimming.

Afterwards a behaviour dependant measure was taken. The participants were promised that they would be rewarded with a freely selected refreshment. They could select only one product from a list containing items such as chocolate, apples, carrots, cakes, grapefruits, and crisps. Upon writing down their choice on a separate sheet of paper, the participants expected to be offered the chosen refreshment. Thus the intention to eat the high-calorie or dietary snack that was recorded by the respondents on the sheet of paper was treated as a behaviour dependent measure.

After completing the experiment, the participants were thanked and fully debriefed. They were informed that only low-calorie items (i.e., apples, carrots, and grapefruits) were available.

### 3.2. Results

The hypothesis was verified on the basis of an analysis of variance. We used the design gender (2) × group (3) to explain the variance of the dependent variable-food choice. No main gender effect or interaction effect was observed.

#### Verification of Hypotheses 2 and 3

Hypotheses 2 and 3 pertained to the influence of priming on the choice of food by individuals from the experimental groups. We assumed that participants from both experimental groups would choose the healthy options more frequently than would the controls.

We constructed a *z*-score from the dependent variable. Analysis of variance revealed a significant effect of group on food choice: *F*(2.96) = 3.98, *p* < 0.03, *eta*^2^ = 0.08, with a significant difference between food choices made by individuals exposed to fattening food *M* = 0.23 versus neutral images *M* = −0.37, *t*(66) = 2.37, *p* < 0.03. Similarly, there was a significance difference between food choice by subjects in experimental group 2 (dietary priming) *M* = 0.14 versus controls *M* = −0.37, *t*(66) = 2.0, *p* = 0.05. The results confirmed both tested hypotheses.

**Figure 2:**
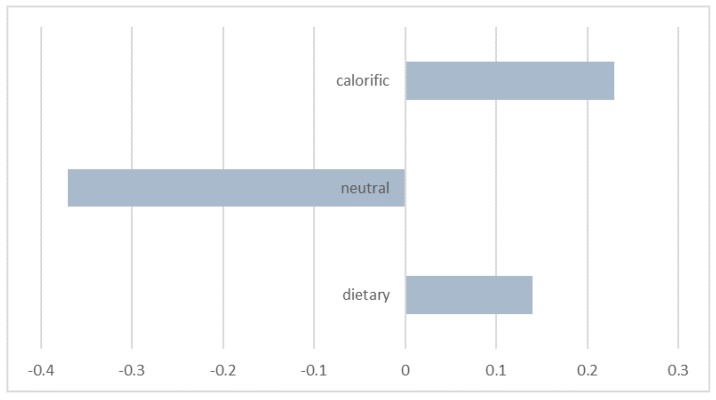
The role of priming on pro-healthy food choice among obese elderly.

Fattening and healthy stimuli acted in the same direction for the participants’ choices of dietary snack (*t*(66) = −0.46, n.s.). Priming both healthy and fattening food in obese participants resulted in healthier food choice in contrast to individuals exposed to neutral priming (Figure 2).

### 3.3. Discussion

Very often seniors are overweight, unfortunately, their weight concerns are not sufficiently reflected by a higher level of health-seeking behaviours [42]. The aim of this study was to verify the effect of priming a stimulus endangering, and consistent with, the higher-priority goal (i.e., losing weight) in obese elderly attempting to lose weight. We verified whether the strategies of self-control acquired during the slimming program, as well as the involvement of our participants in the realization of their goals, proved strong enough to develop an asymmetrically facilitating association between the higher-priority goal and temptation, as well as strengthening the association between the stimulus consistent with the realization of the plan and the plan itself. The presence of such relationships between external stimuli, both consistent and inconsistent with the higher-priority goal, significantly modulates the effectiveness of dietary self-control [35,43].

Our hypothesis, according to which individuals primed for both fattening and healthy food should choose the healthy option more frequently than the controls did, was confirmed. The real response to endangering, as well as encouraging, stimuli suggests that an external stimulus which is consistent with the higher-priority goal is reflected by the consistent behaviour of obese individuals who are involved with and actively practicing proper dietary habits.

Moreover, of special concern was the second hypothesis, which concerned the behavioural aspect of self-regulation, i.e., self-control over the temptation of fattening food. We assumed that subjects primed for fattening food would choose healthy snacks more often than the controls did. This assumption was based on the theoretical background as well as on the findings of Study I. This study confirmed an asymmetrically facilitating association between temptation and the higher-priority goal of slimming in obese subjects. Therefore, we assumed that due to the self-control mechanism activated by the endangering stimulus, our subjects would be better protected against the real risks associated with fattening food than the controls were. Our findings confirmed this prediction. Our findings also supported results obtained in similar research on the impact of priming on dietary self-control [43,44,45]. This, again, suggests that a high level of motivation and active involvement in learning, implementing, and practicing proper nutritional habits leads to the development of intrinsic control mechanisms.

## 4. Practical Implications of These Studies

Obesity is an acute medic problem in elderly as we pointed out in the introduction. Moreover, obesity threatens the psychophysical health of the elderly. According to the study, abdominal obesity especially widespread among above population, is particularly associated with cardiovascular and metabolic diseases [46,47]. Besides it affects body-esteem and therefore self-esteem of obese people [48]. That is why many people especially women decide to lose weight. However observations of the slimming patients show that they are struggling with various psychosocial problems while body reduction [39]. For instance obese individuals who wish to modify their dietary habits to permanently reduce their bodyweight are especially afraid of the risks associated with contact with fattening food [49]. They worry they will not be able to overcome the temptation to eat. So they stop meeting their families and friends to avoid exposure to high-calorie food [50]. They fall into conflict with their housemates, whose dietary habits they try to modify to eliminate tempting food products from their immediate surroundings [51]. If continued for longer, such situations are reflected by psychological discomfort and can lead them to believe that their new dietary habits represent too radical a modification, one which negatively affects their relationships with others, and generally makes them less happy [51].

Not infrequently, individuals with excess body weight who undertake inefficient attempts to lose weight claim that their own internal predisposition, which is almost impossible to change, is the main reason behind their failure [49,50]. They declare that they would not experience such difficulties controlling their dietary habits if they had been born with a sufficiently strong will. They dream of being charmed or given a magic pill to strengthen their weak will, to make them resistant to the temptation of fattening food. Therefore, they focus on mal-adaptive and non-natural methods of losing weight [49,50]. Obviously, a strong will has a basis in predisposition; for example, it can be determined by temperamental factors, such as reactivity or endurance [52,53,54]. However, the realization of our goals is also significantly modulated by our experience and the strategies we employ. This is not infrequently associated with changes which can initially cause a certain degree of psychophysical discomfort until one becomes used to them. The second experiment proved that a strong will, something demanded by so many obese individuals who are ineffective at slimming, can be effectively improved by one’s involvement with and systematic training of proper dietary habits [55]. As a result, such individuals can develop a facilitating association between external stimuli which are consistent with or endanger the higher-priority goal of losing weight. For others, such an association makes them resistant to temptations which remain out of their control. Moreover, contact with fattening food can itself be a motivation to lose weight.

## 5. Limitations of the Studies

It should be understood that the priming procedure is a rather weak experimental manipulation. According to many experimental psychologists, the same kind of priming can cause completely different effects. Drouin and Davidson [55], for instance, showed that priming money might result in limitations of working memory, or in the enhancement of working memory. On the other hand, there is substantial evidence for priming effects in food advertising affecting eating behaviour. Harris et al. [56] showed that watching advertisements for fattening food resulted in an increase of high-calorie food consumption among children (>45%), and a similar increase in consumption of healthy as well as high-calorie snacks among adults. The authors proclaimed that “advertising is a ‘real-world’ prime” [56]. They insist that priming food really works. Bearing in mind the limitations of priming as an experimental manipulation, it can be proposed that priming unhealthy food really works and can lead to negative consequences.

Moreover, the most severe limitation of our study is the declarative nature of the measures of motivation to diet. Only two items were used, separately, to assess the need for the *improvement of one’s silhouette* and *losing excess weight.* No attempt was made to create a questionnaire that measures motivation to diet. Future research on that subject could be additionally developed by introducing participants as young as 50 years old to the experiments. As well extending type and scope of experimental stimuluses’ in study could possibly yield valuable data. Besides elderly people are more likely to experience physical illness or personal loss which are contributing factors to anxiety and depression [57]. Also different types of emotional crises and internal conflicts are related to the process of aging [57]. Therefore further work and new research should also analyze impact of mental condition of respondents on the effects of the conscious processing of stimuli associated with dietary habits. The results of our studies should be treated with especial prudence.

## 6. Conclusions

In our study we verified whether the strategies of self-control acquired during the slimming program, and the involvement of our obese elderly participants in learning, implementing, and practicing proper nutritional habits proved strong enough to develop an asymmetrically facilitating association between the higher-priority goal and temptation. Our study revealed that while looking at fattening food constitutes a significant threat for non-slimming obese elderly, it is not dangerous for actively slimming obese persons, i.e., those who practice effective strategies of self-control and feel self-motivated to lose weight. The latter serves as a proof for counteractive control. High level of motivation and active involvement in the realization of the goals leads to the development of intrinsic control mechanisms. Individuals who are motivated and involved in achieving a given goal utilize anti-temptation strategies. Therefore, sticking to the rules while making changes necessary for losing weight is sufficient to protect against the dangers of contact with high-calorie food. The results of above study constitute important theoretical inputs, and they could contribute to the effectiveness of psychological interventions in obesity treatment.
